# Is the Linear Modeling Technique Good Enough for Optimal Form Design? A Comparison of Quantitative Analysis Models

**DOI:** 10.1100/2012/689842

**Published:** 2012-11-11

**Authors:** Yang-Cheng Lin, Chung-Hsing Yeh, Chen-Cheng Wang, Chun-Chun Wei

**Affiliations:** ^1^Department of Arts and Design, National Dong Hwa University, Hualien 974, Taiwan; ^2^Faculty of Information Technology, Monash University, Clayton, VIC 3800, Australia; ^3^Department of Computer Simulation and Design, Shih Chien University, Kaohsiung 845, Taiwan; ^4^Department of Industrial Design, National Cheng Kung University, Tainan 701, Taiwan

## Abstract

How to design highly reputable and hot-selling products is an essential issue in product design. Whether consumers choose a product depends largely on their perception of the product image. A consumer-oriented design approach presented in this paper helps product designers incorporate consumers' perceptions of product forms in the design process. The consumer-oriented design approach uses quantification theory type I, grey prediction (the linear modeling technique), and neural networks (the nonlinear modeling technique) to determine the optimal form combination of product design for matching a given product image. An experimental study based on the concept of Kansei Engineering is conducted to collect numerical data for examining the relationship between consumers' perception of product image and product form elements of personal digital assistants (PDAs). The result of performance comparison shows that the QTTI model is good enough to help product designers determine the optimal form combination of product design. Although the PDA form design is used as a case study, the approach is applicable to other consumer products with various design elements and product images. The approach provides an effective mechanism for facilitating the consumer-oriented product design process.

## 1. Introduction

Products have been considered a symbol of occupation, personality, opinion, and other human attributes. Whether a product is successful largely depends on the final judgment of consumers [1]. Therefore, product designers need to comprehend the consumers' needs in order to design successful products (highly-reputable and hot-selling) in an intensely competitive market [2]. Moreover, a successful product should not only possess good functionalities, interface design, and operating performance, but also need to take the product image design into account to satisfy consumers' psychological requirements [3]. The external appearance of a product can represent a product image that evokes consumers' internal resonance and consuming motivation [4]. The product image engages an influential factor in consumers' preference structure [5]. When choosing a product, consumers tend to rely on their own particular perception of the product, which is regarded as something of a black box [6]. As an ergonomic consumer-oriented methodology, Kansei Engineering is developed as integrative design strategies for affective design to satisfy consumers' psychological requirements [7–9]. The word “Kansei” indicates the consumers' psychological requirements or emotional feelings of a product. Kansei Engineering has been used to assist product designers in designing product forms that can best match specific product images [10, 11].

In this paper, we present a consumer-oriented design approach addressing for challenging issues in designing consumer products, such as personal digital assistants (PDAs). What are the key form elements for a desirable product image? How to use the adequate product form combination to enhance consumers' preference? Is there an optimal combination of product form that best matches a desirable feeling of the consumers? For example, if product designers want to design a product with “simple-to-look” appearance, are there guidelines of product form design to follow? In addition, nonlinear modeling techniques (such as the artificial intelligent system or the soft computing) are defined as “an emerging approach to reasoning and learning the human mind in an uncertainty and imprecision environment” [12, 13]. These techniques are supposed to possess humanlike expertise within a specific domain, adapt themselves and learn to do better in changing environments, and explain how they make decisions [9, 12]. Hence, are the nonlinear modeling techniques suitable for exploring the relationship between the consumers' perceptions of product images and product form elements? Or are the linear modeling techniques good enough to do so [14]? What specific technique should be used to help product designers determine the optimal form combination of product design for a particular design concept of product image? To illustrate how the approach can be used to answer these research questions, we conduct an experimental study on PDAs, using two linear modeling techniques and one nonlinear modeling technique. Two linear modeling techniques are the quantification theory type I (QTTI) [15] and the grey prediction (GP) [16], and the nonlinear modeling technique is the neural networks (NNs) [17].

The QTTI is a variant of linear multiple regression analysis and can be used to quantify the relationships between product form elements and product images [5], while the GP model can deal with incomplete information effectively and requires only four data sets or more [16]. As such, the GP can be used to predict how a particular combination of product form elements matches a product image, particularly when the information is available only for a limited number of product form elements [10]. Due to the effective learning ability, NNs have been applied successfully in a wide range of fields, using various learning algorithms [18–20]. NNs are well suited to formulate the product design process for matching the product form (the input variables) to the consumers' perceptions (the output variables), which is often a black box and cannot be precisely described [10].

In subsequent sections, we first present the quantitative analysis methods used to analyze the experimental data sets for answering the research questions. Then we conduct an experimental study on PDAs to describe how Kansei Engineering can be used to extract representative samples and product form elements as numerical data sets required for analysis. Finally, we discuss the results of applying these techniques and evaluate their performance in order to determine the better model that can be used to help product designers meet consumers' requirements for a desirable product image.

## 2. Methods of Quantitative Analysis

In this section, we present a brief outline of the relevant theories and algorithms, including the QTTI, the GP, and the NNs. We use these techniques to examine the relationship between product form elements and product images.

### 2.1. Quantification Theory Type I

The QTTI can be regarded as a method of qualitative and categorical multiple regression analysis method [15], which allows inclusion of independent variables that are categorical and qualitative in nature, such as product form elements and quantitative criterion variables within Kansei Engineering. In Kansei Engineering, product form elements are typically classified into two levels that correspond to form design element and its treatments, respectively. The QTTI consists of the followings six steps [15].

**Step 1**

Define the Kansei relational model associated with the Kansei measurement scores of experimental samples with respect to an image word pair.

In Kansei Engineering, the criterion variables represent the product image, and the explanatory variables represent the product form elements. The categorical multiple regression model can be defined as

(1) $$\begin{array}{c} {\hat{y}}_{s}^{k}=\sum_{i=1}^{E}\sum_{j=1}^{C_{i}}\beta_{ij}x_{ijs}+\varepsilon , \end{array}$$

where ${\hat{y}}_{s}^{k}$: the predicted value of the criterion variable for the *s*th product sample on the *k*th image word; *i*: the index of design element, *E*: the number of design element; *j*: the index of category; *C*_*i*_: the number of category of the *i*th design element; *ε*: a stochastic variable whose expectation value *E*(*ε*) = 0; *β*_*ij*_: the category score of the *j*th style within the *i*th design element; *x*_*ijs*_: the coefficient of the dummy variable that is the explanatory variable or the dummy variable representing the *j*th style within the *i*th design element using the *s*th experimental sample.

**Step 2**

Calculate the standardized regression coefficients and the standardized constant in the model. The model of categorical multiple regression analysis can be redefined as

(2) $$\begin{array}{c} {\hat{y}}_{s}^{k}=\sum_{i=1}^{E}\sum_{j=1}^{C_{i}}\beta_{ij}^{*}x_{ijs}+{\overset{-}{y}}_{s}^{k},\\ \beta_{ij}^{*}=\beta_{ij}-\frac{1}{n}\sum_{j=1}^{C_{i}}\beta_{ij}x_{ijs},\\ {\overset{-}{y}}_{s}^{k}=\frac{1}{n}\sum_{s=1}^{n}y_{s}^{k}, \end{array}$$

where *β*_ij_* represents the standardized coefficient of explanatory variables and ${\bar{y}}_{s}^{k}$ is the standardized constant in the model.

**Step 3**

Determine the matrix CCR of correlation coefficient of all variables.

**Step 4**

Calculate the multiple correlation coefficient *R* that is regarded as the relational degree of external criterion variable and explanatory variables.

**Step 5**

Calculate the partial correlation coefficients (PCC) of design elements to clarify the relationships between product form elements and a product image.

**Step 6**

Determine the statistical range of a categorical variable (product form element) by the difference between the maximum value and minimum value of the category score. The range of the categorical variable indicates its contribution degree to the prediction model with respect to a given product image.

### 2.2. Grey Prediction

The grey system theory [16] has been developed to examine the relationship among factors in an observable system where the information available is grey, meaning uncertain and incomplete (i.e., only part of the information is known). It has been successfully used in a wide range of fields, including some recent application results [10, 21–23] highlighting its effective handling of incomplete known information for exploring unknown information. The system that can be built for answering specific research questions in product design with respect to product form and product image is grey in essence, as there is no way to identify all the product form elements that affect a particular product image perceived by consumers [10].

The GP model uses a grey differential model (GM) to generate data series from the original data series of a dynamic system. The data series generated by the GM are converted back to the original data series by a reverse procedure to predict the performance of the system. Since the generated data series are more coherent than the original, the accuracy of the modeling is enhanced. The GM has three basic operations [16]: (1) accumulated generation, (2) inverse accumulated generation, and (3) grey modeling. The accumulated generation operation (AGO) is used to build differential equations. The GM is usually represented as GM(*M*, *N*) for dealing with *M*th-order differential equations with *N* variables. Since any higher-order differential equation can be transferred into a first-order differential equation, we use the first-order differential equation in this paper.

The GM(1, 1), a single variable and first-order grey model, is one of the most frequently used grey prediction models. Its procedure involves the following four steps.

**Step 1**

Denote the original sequence as

(3) $$\begin{array}{c} x^{\left(0\right)}=\left(x^{\left(0\right)}\left(1\right),x^{\left(0\right)}\left(2\right),\ldots ,x^{\left(0\right)}\left(n\right)\right), \end{array}$$

where *x* ^(0)^(*i*) is the time series data at time *i*  (*i* = 1,2,…, *n*).

**Step 2**

Generate a new sequence *x* ^(1)^ by the AGO based on the original sequence *x* ^(0)^, where

(4) $$\begin{array}{c} x^{\left(1\right)}=\left(x^{\left(1\right)}\left(1\right),x^{\left(1\right)}\left(2\right),\ldots ,x^{\left(1\right)}\left(n\right)\right), \end{array}$$

(5) $$\begin{array}{c} x^{\left(1\right)}\left(1\right)=x^{\left(0\right)}\left(1\right),\;\;x^{\left(1\right)}\left(k\right)=\sum_{i=1}^{k}x^{\left(0\right)}\left(i\right). \end{array}$$

**Step 3**

Define the first-order differential equation as

(6) $$\begin{array}{c} \frac{dx^{\left(1\right)}}{dt}+ax^{\left(1\right)}=b. \end{array}$$

**Step 4**

Use the least square method to solve (4) by

(7) $$\begin{array}{c} {\hat{x}}^{\left(1\right)}\left(k+1\right)=\left(x^{\left(0\right)}\left(1\right)-\frac{b}{a}\right)e^{-ak}+\frac{b}{a},\\ {\hat{x}}^{\left(0\right)}\left(k+1\right)={\hat{x}}^{\left(1\right)}\left(k+1\right)-{\hat{x}}^{\left(1\right)}\left(k\right), \end{array}$$

where

(8) $$\begin{array}{c} \hat{a}=\left[\begin{array}{c} a\\ b \end{array}\right]={\left(B^{T}B\right)}^{-1}B^{T}y_{1},\\ B=\left[\begin{array}{cc} -0.5\left(x^{\left(1\right)}\left(1\right)+x^{\left(1\right)}\left(2\right)\right) & 1\\ -0.5\left(x^{\left(1\right)}\left(2\right)+x^{\left(1\right)}\left(3\right)\right) & 1\\ \vdots & \vdots \\ -0.5\left(x^{\left(1\right)}\left(n-1\right)+x^{\left(1\right)}\left(n\right)\right) & 1 \end{array}\right],\\ y_{1}={\left(x^{\left(0\right)}\left(2\right),x^{\left(0\right)}\left(3\right),\ldots ,x^{\left(0\right)}\left(n\right)\right)}^{T}. \end{array}$$

The ${\hat{x}}^{\left(1\right)}\left(k+1\right)$ is the predicted value of *x* ^(1)^(*k* + 1) and ${\hat{x}}^{\left(0\right)}\left(k+1\right)$ is the predicted value of *x* ^(0)^(*k* + 1) at time *k* + 1. We can also use the inverse accumulated generation operation (IAGO) to obtain ${\hat{x}}^{\left(0\right)}\left(k+1\right)$ as

(9) $$\begin{array}{c} {\hat{x}}^{\left(0\right)}\left(k+1\right)=\left(x^{\left(0\right)}\left(1\right)-\frac{b}{a}\right)\left(1-e^{a}\right)e^{-ak}. \end{array}$$

The GM(1,1) grey model can be extended to the GM(1, *N*) model [10, 16], first-order with *N* variables (*x*_1_^(0)^, *x*_2_^(0)^, *x*_3_^(0)^,…, *x*_*N*_^(0)^). The differential equation can be defined as

(10) dx1(1)dt+ax1(1)=b1x2(1)+b2x3(1)+⋯+bN−1xN(1)=∑i=2Nbi−1xi(1),

where *a*, *b*_1_, *b*_2_,…, *b*_*N*−1_ are unknown parameters and can be calculated by

(11) $$\begin{array}{c} \hat{a}=\left(a,b_{1},b_{2},\ldots ,b_{N-1}\right)={\left(B^{T}B\right)}^{-1}B^{T}y_{N}, \end{array}$$

where

(12) $$\begin{array}{c} B=\left[\begin{array}{ccc} -0.5\left({x_{1}}^{\left(1\right)}\left(1\right)+{x_{1}}^{\left(1\right)}\left(2\right)\right) & {x_{2}}^{\left(1\right)}\left(2\right) & \cdots {x_{N}}^{\left(1\right)}\left(2\right)\\ -0.5\left({x_{1}}^{\left(1\right)}\left(2\right)+{x_{1}}^{\left(1\right)}\left(3\right)\right) & {x_{2}}^{\left(1\right)}\left(3\right) & \cdots {x_{N}}^{\left(1\right)}\left(3\right)\\ \vdots & \vdots & \vdots \\ -0.5\left({x_{1}}^{\left(1\right)}\left(n-1\right)+{x_{1}}^{\left(1\right)}\left(n\right)\right) & {x_{2}}^{\left(1\right)}\left(n\right) & \cdots {x_{N}}^{\left(1\right)}\left(n\right) \end{array}\right],\\ y_{N}={\left(x^{\left(0\right)}\left(2\right),x^{\left(0\right)}\left(3\right),x^{\left(0\right)}\left(4\right),\ldots ,x^{\left(0\right)}\left(n\right)\right)}^{T}. \end{array}$$

The prediction of *x*_1_^(1)^ is defined as

(13) x^1(1)(k+1)=(x1(0)(1)−∑i=2Nbi−1axi(1)(k+1))e−ak +∑i=2Nbi−1axi(1)(k+1).

The ${\hat{x}}^{\left(1\right)}\left(k+1\right)$ is the predicted value of *x* ^(1)^(*k* + 1) of the GM(1, *N*) at time *k* + 1.

### 2.3. Neural Networks

NNs are nonlinear models and are widely used to examine the complex relationship between input variables and output variables [17]. In this paper, we use the multilayered feedforward neural networks trained with the backpropagation learning algorithm, as it is an effective and the popular supervised learning algorithm [10].

A typical three-layer network consists of an input layer, an output layer, and one hidden layer, with *n*, *m*, and *p* neurons, respectively (indexed by *i*, *j*, and *k*, resp.) [24]. The *w*_*ij*_ and *w*_*jk*_ represent the weights for the connection between neuron *i*  (*i* = 1,2,…, *n*) and neuron *j*  (*j* = 1,2,…, *m*), and between neuron *j*  (*j* = 1, 2, …, *m*) and neuron *k*  (*k* = 1, 2, …, *p*), respectively. In training the network, a set of input patterns or signals, (*x*_1_, *x*_2_,…, *x*_*n*_) is presented to the network input layer. The network then propagates the inputs from layer to layer until the outputs are generated by the output layer. This involves the generation of the outputs (*y*_*j*_) of the neurons in the hidden layer as given in (14) and the outputs (*y*_*k*_) of the neurons in the output layer as given in (15).

(14) $$\begin{array}{c} y_{j}=f\left(\sum_{i=1}^{n}x_{i}w_{ij}-\theta_{j}\right), \end{array}$$

(15) $$\begin{array}{c} y_{k}=f\left(\sum_{j=1}^{m}x_{j}w_{jk}-\theta_{k}\right), \end{array}$$

where *f*(·) is the sigmoid activation function as given in (16), and *θ*_*j*_ and *θ*_*k*_ are threshold values:

(16) $$\begin{array}{c} f\left(X\right)=\frac{1}{1+e^{-X}}. \end{array}$$

If the outputs (*y*_*k*_) generated by (15) are different from the target outputs (*y*_k_*), errors (*e*_1_, *e*_2_,…, *e*_*p*_) are calculated by (17) and then propagated backwards from the output layer to the input layer in order to update the weights for reducing the errors.

(17) $$\begin{array}{c} e_{k}=y_{k}^{*}-y_{k}. \end{array}$$

The weights (*w*_*jk*_) at the output neurons are updated as *w*_*jk*_ + Δ*w*_*jk*_, where Δ*w*_*jk*_ is computed by (known as the delta rule)

(18) $$\begin{array}{c} \Delta w_{jk}=\alpha y_{j}\delta_{k}, \end{array}$$

where *α* is the learning rate (usually 0 < *α* ≤ 1) and *δ*_*k*_ is the error gradient at neuron *k*, given as

(19) $$\begin{array}{c} \delta_{k}=y_{k}\left(1-y_{k}\right)e_{k}. \end{array}$$

The weights (*w*_*ij*_) at the hidden neurons are updated as *w*_*ij*_ + Δ*w*_*ij*_, where Δ*w*_*ij*_ is calculated by

(20) $$\begin{array}{c} \Delta w_{ij}=\alpha x_{i}\delta_{j}, \end{array}$$

where *α* is the learning rate (usually 0 < *α* ≤ 1) and *δ*_*j*_ is the error gradient at neuron *j*, given as

(21) $$\begin{array}{c} \delta_{j}=y_{j}\left(1-y_{j}\right)\sum_{k=1}^{p}\delta_{k}w_{jk}. \end{array}$$

The training process is repeated until a specified error criterion is satisfied.

## 3. Experimental Procedures of Consumer-Oriented Design

We conduct an experimental study using the concept of Kansei Engineering in order to collect numerical data about the relationship between product form elements and a given product image of PDAs. The experimental study involves three main steps: (a) extracting representative experimental samples, (b) conducting morphological analysis of product form elements, and (c) assessing consumers' perceptions for a given product image.

### 3.1. Extracting Representative Experimental Samples

In the experimental study, we investigate and categorize various PDAs on the market. We first collect 88 PDAs and then classify them based on their similarity degree. To collect opinions regarding the usage, function, and form of PDAs, a focus group is formed by six subjects with at least two years' experience of using the PDA. The focus group eliminates some highly similar samples through discussions. Then the *K*-means cluster analysis is used to extract representative samples of PDAs. There are 30 representative PDA samples, including 24 samples as the training set and six samples as the test set for building quantitative models in Section 4.

### 3.2. Conducting Morphological Analysis of Product Form Elements

The product form is defined as the collection of design features that the consumers will appreciate. The morphological analysis [25], concerning the arrangement of objects and how they conform to create a whole of Gestalt, is used to explore all possible solutions in a complex problem regarding a product form.

The morphological analysis is used to extract the product form elements of the 30 representative samples. The six subjects of the focus group are asked to decompose the PDA samples into several dominant form elements and form types according to their knowledge and experience.  Table 1 shows the result of the morphological analysis, with six product design elements (i.e., top shape, bottom shape, function-keys arrangement, arrow-key style, color treatment, and outline partition style) and 19 associated product form types being identified. The form type indicates the relationship between the outline elements. For example, the “top shape (*X*_1_)” form element has three form types, including “line (*X*_11_),” “chamfer (*X*_12_),” and “fillet (*X*_13_).” A number of design alternatives can be generated by various combinations of morphological elements [26].

### 3.3. Assessing Consumers' Perceptions of Product Images

In Kansei Engineering, image assessment experiments are usually performed to elicit the consumers' psychological feelings or perceptions about a product using the semantic differential method. Pairs of image words are often used to describe the consumers' perceptions of the product in terms of ergonomic and psychological estimation. With the identification of the form elements of the product, the relationship between the image words and the form elements can be established.

In this paper, the image word pair used for representing the product image of PDAs is Simple-Complex (S-C) about the visibility aspect, according to our previous study [27]. In Wang et al. [27], we use these 30 representative PDA samples and product images to examine whether the NN model is an effective technique and what structure is better for the product form design among 4 NN models built with different hidden layer neurons. In this study, we use the same experimental data as a basis for addressing new and significant research issues as stated in Section 1.

To obtain the assessed values for the product image of 30 representative PDA samples, a 10-point scale (1–10) of the semantic differential method is used. 52 subjects (30 males and 22 females with ages ranging from 26 to 45, mean = 35.4, SD = 4.4) are asked to assess the form (look) of PDA samples on a simplicity-complexity scale of 1 to 10, where 10 is most simple and 1 is most complex. The last column of Table 2 shows the assessed S-C value of the 30 PDA samples, including 24 samples in the training set and six samples in the test set (asterisked). For each selected PDA in Table 2, the first column shows the PDA number and Columns 2–7 show the corresponding type number for each of its six product form elements, as given in Table 1. Table 2 provides a numerical data source for building quantitative models, which can be used to develop a design support system for simulating the optimal form design process for PDAs.

## 4. Experimental Analysis and Results 

In this section, we present the results of applying the QTTI, the GP, and the NN models in order to explore the relationship between product form elements and consumers' perceptions for a given product image, using the assessing results summarized in Tables 1 and 2.

### 4.1. The QTTI Analysis and Results

We use the QTTI analysis to examine the relationship between the six product form elements and the S-C product image. In this paper, six independent variables (i.e., the six product form elements) and one dependent variable (i.e., the S-C product image) are used. The result of QTTI analysis is given in Table 3. In Table 3, the partial correlation coefficients indicate the relationship between the six product form elements (*X*_1_, *X*_2_, *X*_3_, *X*_4_, *X*_5_, and *X*_6_) and the S-C product image (*Y*). The highest variable of the partial correlation coefficient in the “S-C” image is the “arrow-key style” form element (*X*_4_ = 0.42), meaning that “arrow-key style” primarily affects the “S-C” image of the product, followed by the “color treatment” form element (*X*_5_ = 0.37) and the “top shape” form element (*X*_1_ = 0.26). This implies that the product designers should focus their attention more on these most influential elements, when the objective of designing a new PDA is to achieve a desirable “S-C” image. On the contrary, the product designers can pay less attention to the less influential elements such as “bottom shape” form element (*X*_2_ = 0.14), and the “function-keys arrangement” form element (*X*_3_ = 0.16), as these form elements contribute relatively little to the consumers' perceptions of the “S-C” image on the PDAs.

In the last second row of Table 3, *R* means the correlation between the observed and predicted values of the dependent variable, and *R* ^2^ is the square of this correlation. *R* ^2^ ranges from 0 to 1. If there is no linear relation between the dependent variable (*Y*) and independent variables (*X*_1_, *X*_2_, *X*_3_, *X*_4_, *X*_5_, and *X*_6_), *R* ^2^ is 0 or very small. Otherwise, if all the values fall on the regression line, *R* ^2^ is 1. The category grade (form type grade) shown in Table 3 indicates the preference degree of the consumers' perception on each category of independent variables. If the grade is negative, the consumers' perception leans towards the “complex” image. On the contrary, the positive grade indicates that the consumers' perception favors the “simple” image. For example, the category grades of 3 selected values of “outline partition style (*X*_6_)” in the “S-C” image are −0.20, −0.13, and 1.32, respectively. The result shows that the consumers' perception prefers the “complex” image if the “outline partition style (*X*_6_)” is “normal partition (*X*_61_)” or “fitting outline (*X*_62_),” and favors the “simple” image while “outline partition style (*X*_6_)” is “fitting surface (*X*_63_).”

As the result of the QTTI analysis, Model (22) indicates the relationship between product form elements and the S-C product image. We can use this model to input the values of six product form variables, and then output the predicted value of the S-C product image. This model can help the product designers understand consumers' perceptions to find out the optimal combination of product form design in terms of a given product image:

(22) y^=3.74+X11+0.54X12−0.42X13−0.11X21+0.61X22 −0.19X23+0.01X31−0.37X32+0.65X33+0.48X34 −0.42X41+1.28X42−1.29X43−0.21X51+1.35X52 −1.06X53−0.20X61−0.13X62+1.32X63.

### 4.2. The GP Analysis and Results

The GP is used as a technique for determining the optimal combination of product form elements for matching a desirable product image. The 24 samples in the training set, given in Table 2, are used as the data set for building the GP model. 

As a GM(1,7), the GP model uses the six form elements as the comparison series *X*_*i*_ and the average S-C values as the reference series *X*_0_. To build the GP model, we first obtain a new sequence *x* ^(1)^ for each series using (3)–(5) and the AGO as

(23) $$\begin{array}{c} \left(\begin{array}{c} {x_{0}}^{\left(1\right)}\\ {x_{1}}^{\left(1\right)}\\ {x_{2}}^{\left(1\right)}\\ \vdots \\ {x_{6}}^{\left(1\right)} \end{array}\right)=\left(\begin{array}{ccccc} 1.67, & 4.00, & 7.33, & \cdots & 89.66\\ 3, & 6, & 8, & \cdots & 60\\ 1, & 2, & 4, & \cdots & 53\\ \cdots & \cdots & \cdots & \cdots & \cdots \\ 1, & 2, & 4, & \cdots & 33 \end{array}\right). \end{array}$$

We then apply (10)–(12) to obtain the parameters of $\hat{a}$ as

(24) $$\begin{array}{c} \hat{a}=\left(\begin{array}{c} a\\ b_{1}\\ b_{2}\\ b_{3}\\ b_{4}\\ b_{5}\\ b_{6} \end{array}\right)=\left(\begin{array}{c} 0.38\\ -0.25\\ -0.29\\ -0.60\\ 0.69\\ 1.13\\ -0.36 \end{array}\right). \end{array}$$

The GP model for predicting the S-C value based on the six form elements is thus built by (13) as

(25) X^0(1)(k+1)=[1.67+6.46X1(1)(k+1)+7.68X2(1)(k+1)    +15.67X3(1)(k+1)−17.92X4(1)(k+1)     −29.52X5(1)(k+1)+9.51X6(1)(k+1)]e−0.038k −6.46X1(1)(k+1)−7.68X2(1)(k+1) −15.67X3(1)(k+1)+17.92X4(1)(k+1) +29.52X5(1)(k+1)−9.51X6(1)(k+1).

With the GP model in (25), product designers can input the value of the corresponding form elements, and then obtain a predicted S-C value.

### 4.3. The NN Analysis and Results

To examine whether the NN model is an effective technique for determining the optimal combination of product form elements for matching a desirable product image, we develop two neural network models, called NN-FE and NN-FT, respectively. The NN-FE uses all the six form elements (FE) as input variables (input neurons), while the NN-FT has 19 input neurons, which are the whole 19 form types (FT) of the six form elements identified from the experimental study. For the NN-FE model, if a PDA has a particular type of form element, the value of the corresponding input neuron is 1, 2, 3, or 4. On the other hand, for the NN-FT model, if a PDA has a particular type of form element, the value of the corresponding input neuron is 1; otherwise the value is 0. Both NN models use a widely used rule [17], (the number of input neurons + the number of output neurons)/2, for determining the number of neurons in the single hidden layer.  Table 4 shows the neurons of these two NN models, including the input layer, the hidden layer, and the output layer. The learning rule used is Delta-Rule and the transfer function is Sigmoid [17] for all layers. All of input and output variables (neurons) are normalized before training. The learning rate is 0.2, and momentum is 0.5, based on our previous study [28].

**Figure 1:**
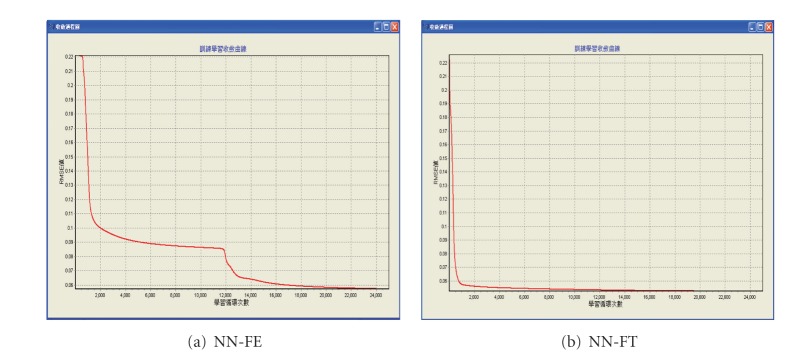
The convergence diagrams of NN-FE and NN-FT in the training process.

The experimental samples are separated into two groups: 24 training samples and six test samples. Each model is trained ten epochs at each run. When the cumulative training epochs are over 25,000, the training process is completed. The root of mean square errors (RMSE) of the NN-FE model is 0.057, while the NN-FT model is 0.052. This result seems to suggest that the number of input neurons and hidden neurons have little influence on the training effect of NN models. However, after further examination, we find out that if more neurons are in the input or hidden layer, the faster the convergence speed becomes (as shown in Figure 1). In other words, if the input layer or hidden layer has more neurons, then the network converges faster. This result suggests that if the input variable has multiple categories (i.e., the qualitative or categorical variable, such as product form elements), the total number of categories (not the number of variables) should be used as the layer neurons.

## 5. Performance Evaluation and Discussion

To evaluate the performance of the QTTI, GP, NN-FE, and NN-FT models developed in this paper in terms of their prediction ability in determining the optimal design combination of PDA form elements for matching a given S-C image, the six samples in the test set given in Table 2 are used.

### 5.1. Performance Comparison

 The second row of Table 5 shows the average S-C values of the six test samples assessed by 52 subjects, which are used as a comparison base for the performance evaluation. With the six test samples as the input, Table 5 shows the corresponding S-C values predicted by using the QTTI (i.e., Model (22)), GP (i.e., Model (25)), NN-FE, and NN-FT models, respectively. To evaluate the performance of a model, the root of mean square errors (RMSE) is commonly used, given as

(26) $$\begin{array}{c} \text{RMSE}=\sqrt{\frac{{\sum_{i=1}^{n}\left(x_{i}-x_{0}\right)}^{2}}{n}}, \end{array}$$

where *X*_*i*_ is the *i*th output value predicted by the model, and *X*_0_ is the expected values assessed by 52 subjects in the experiment. If there is no difference or error between the predicted value and the expected value, the RMSE is 0.

**Figure 2:**
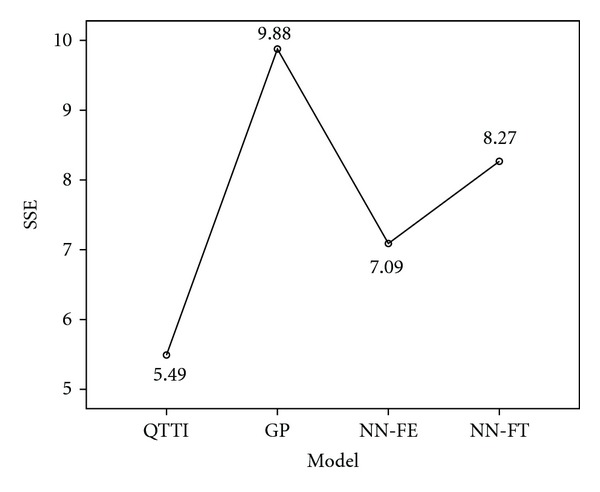
Multiple Comparisons for the SSE of four models.

The last column of Table 5 shows the RMSE of these four models in comparison with the assessed S-C values. Table 5 shows that the lowest RMSE is the QTTI model (0.2343), followed by the NN-FE model (0.2663) and the NN-FT model (0.2875), and the RMSE of the GP model is the highest. The result indicates that the QTTI model has the highest predictive consistency (an accuracy rate of 76.57% = 1 − 0.2343) for predicting the value of the S-C image. This is in line with the result of Multiple Comparisons by one-way Analysis of Variance (one-way ANOVA), as shown in Figure 2. Figure 2 shows the mean of error sum of squares (SSE) for these four models. The lower the SSE, the higher the prediction performance. The result of performance comparison suggests that QTTI is the model to be used for matching a given set of product form elements with a specific product image. 

Nevertheless, this result is not consistent with the common notion that nonlinear quantitative models or systems are more suitable to simulate human beings' thinking and generally have a better performance for predicting consumers' psychological requirements or emotional feelings, in comparison with linear quantitative models [6, 10–12, 18, 29]. In addition, the NN model usually has a better performance and an effective technique to formulate the product design process for determining the optimal combination of product form elements with respect to a desirable product image [5].

### 5.2. Further Evaluation

To further examine the prediction performance of the NN model, we conduct a set of analyses by using different learning rate and momentum factors for getting the better structure of the NN model. Another 3 pairs of learning rate and momentum factors are used for different conditions based on the complication of the research problem. For example, if the research issue is very simple, a large learning rate of 0.9 and momentum of 0.6 are recommended. On more complicated problems or predictive networks where output variables are continuous values rather than categories, use a smaller learning rate and momentum, such as 0.1 and 0.1 respectively. In addition, if the data are complex and very noisy, a learning rate of 0.05 and a momentum of 0.5 are used [30]. To distinguish between the NN-FE and NN-FT models using different input neurons and hidden neurons, both models are associated with the learning rate and momentum mentioned above, such as -P, -S, -C, -N, as shown in Table 6.

**Figure 3:**
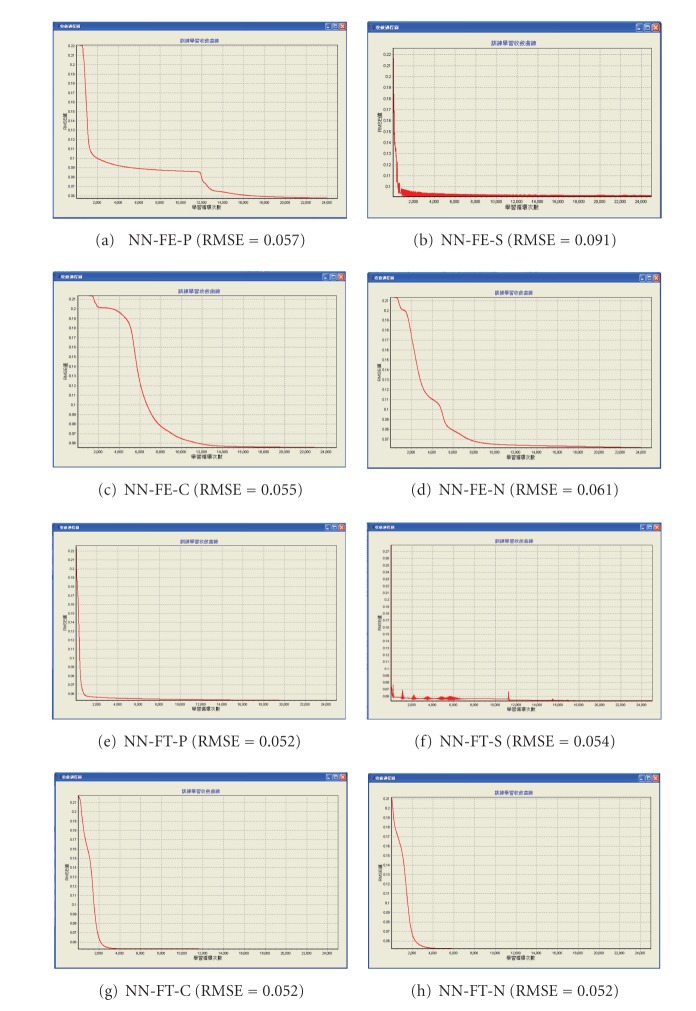
The RMSE and convergence diagrams of NN models in the training process.

As described in Section 4.3, 24 training samples and six test samples are used, and the training process is not stopped until the cumulative training epochs are over 25,000.  Figure 3 shows the RMSE of these eight NN models and the convergence diagrams in the training process. As shown in Figure 3, the convergence speed of NN-FT models are faster than NN-FE models. This is in line with the result of Section 4.3 that the more neurons in the input or hidden layer, the faster the convergence speed. In addition, we find the “-S” models (i.e., NN-FE-S model and NN-FT-S model, both using the large learning rate of 0.9 and momentum of 0.6 if the research issue is very simple) have larger movements as compared to other NN models, thus indicating that the essentials of consumers' perceptions are complicated, and often a block box and cannot be precisely described [10].

With the six test samples as input, Table 7 lists the predicted S-C image values and RMSE of these eight NN models for the further test set. Table 7 shows that the lowest RMSE is the NN-FE-N model (0.2203). In addition, the average RMSE value of NN-FE (0.3033) is slightly smaller than the value of NN-FT (0.3168). This is in line with the result of Section 4.3 that the number of layer neurons (the input or hidden neurons) has little influence on the performing effect of NN models.

### 5.3. Discussion

From the RMSE shown in Table 7, except the NN-FE-N model (the RMSE being 0.2203), the other 7 NN models are larger than the QTTI (the RMSE being 0.2343) shown in Row 3 of Table 5. Further analysis shows that the QTTI model is a better approach for matching a given set of product form elements with a specific product image, regardless of what learning rate and momentum factors are chosen for constructing the NN model. This result implies that the linear modeling technique is good enough to help product designers determine the optimal form combination of product design for a particular design concept of product image. Consequently, in some product design settings, applying nonlinear modeling techniques may not necessarily produce a better outcome. In some settings, the QTTI model (the linear modeling technique) can be used to better explore the relationship between the consumers' perceptions and product form elements without compromising the prediction performance.

According to the experimental analysis and results mentioned above, model (22) can help product designers understand consumers' perceptions of product form for a given product image. This model can also be used to examine the effect of the corresponding product image for a given combination of product form elements. Consequently, the QTTI model enables us to build a PDA design support database that can be generated by inputting each of all possible combinations (972, 3 × 3 × 4 × 3 × 3 × 3) of product form elements to the QTTI model individually for generating the associated image values. Product designers can specify a desirable image value for a new PDA form design, and the database can then work out the optimal combination of form elements.

**Figure 4:**
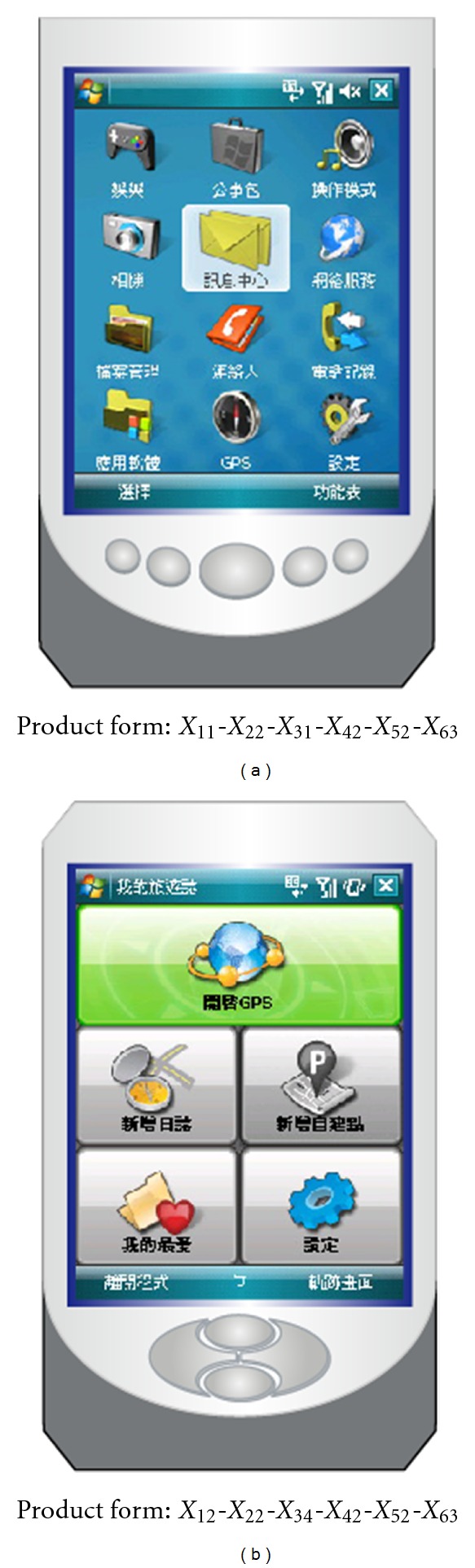
New PDA form designs for the desirable “simple” image.

Table 8 shows the design support information for product designers to find out the optimal combination of product form elements in terms of a given product image. In addition, the design support database can be incorporated into a computer-aided design (CAD) system to facilitate the product form in the new PDA development process. To illustrate, we focus the attention more on the most influential elements, such as the “arrow-key style” form element (*X*_4_) and the “color treatment” form element (*X*_5_), for the desirable “simple” image of PDA.  Figure 4 shows two new PDA form designs with the optimal combination of form elements for the desirable “simple” image.

### 5.4. Limitations and Further Suggestions

In this paper, we use two linear modeling techniques (i.e., quantification theory type I and grey prediction) and one nonlinear modeling technique (i.e., neural networks) to determine the optimal form combination of product design for matching a given product image. In the further studies, other quantitative analysis models should be adopted to test the prediction performance, for example, fuzzy system, genetic algorithm, rough set, multiple regression analysis, and so on. Although PDAs are chosen as the experimental product, the consumer-oriented design approach presented can be applied to other consumer products (e.g., smart phones, Tablet PC, etc.) with various design elements (e.g., color, texture, brand, etc.) and product images (e.g., classic or modern, artificial or artistic, etc.).

## 6. Conclusion

In this paper, we have conducted an experimental study on PDAs to demonstrate how a consumer-oriented design approach can be used to help determine the optimal form combination for matching a given product image. The consumer-oriented design based on the process of Kansei Engineering has used the QTTI model, the grey model, and the neural network model to predict the desirable simple-complex image of consumers' perception. The result of the experimental study has shown that the QTTI model has the highest predictive consistency, thus suggesting that the QTTI model is a better methodological alternative for modeling the consumers' perception of a product characterized by a given set of product form elements. Noteworthily, this result has shown that the QTTI model (the linear modeling technique) is good enough to help product designers determine the optimal form combination of product design for a particular design concept of product image. Consequently, in some product design settings, we can use the linear modeling technique to explore the relationship between the consumers' perceptions and product form elements without compromising the prediction performance. Furthermore, the consumer-oriented design approach has been built a PDA design support database, in conjunction with the computer-aided design (CAD) system, to help product designers facilitate the product form in the new PDA development process.

## Figures and Tables

**Table 1:** Morphological analysis of PDA design forms.

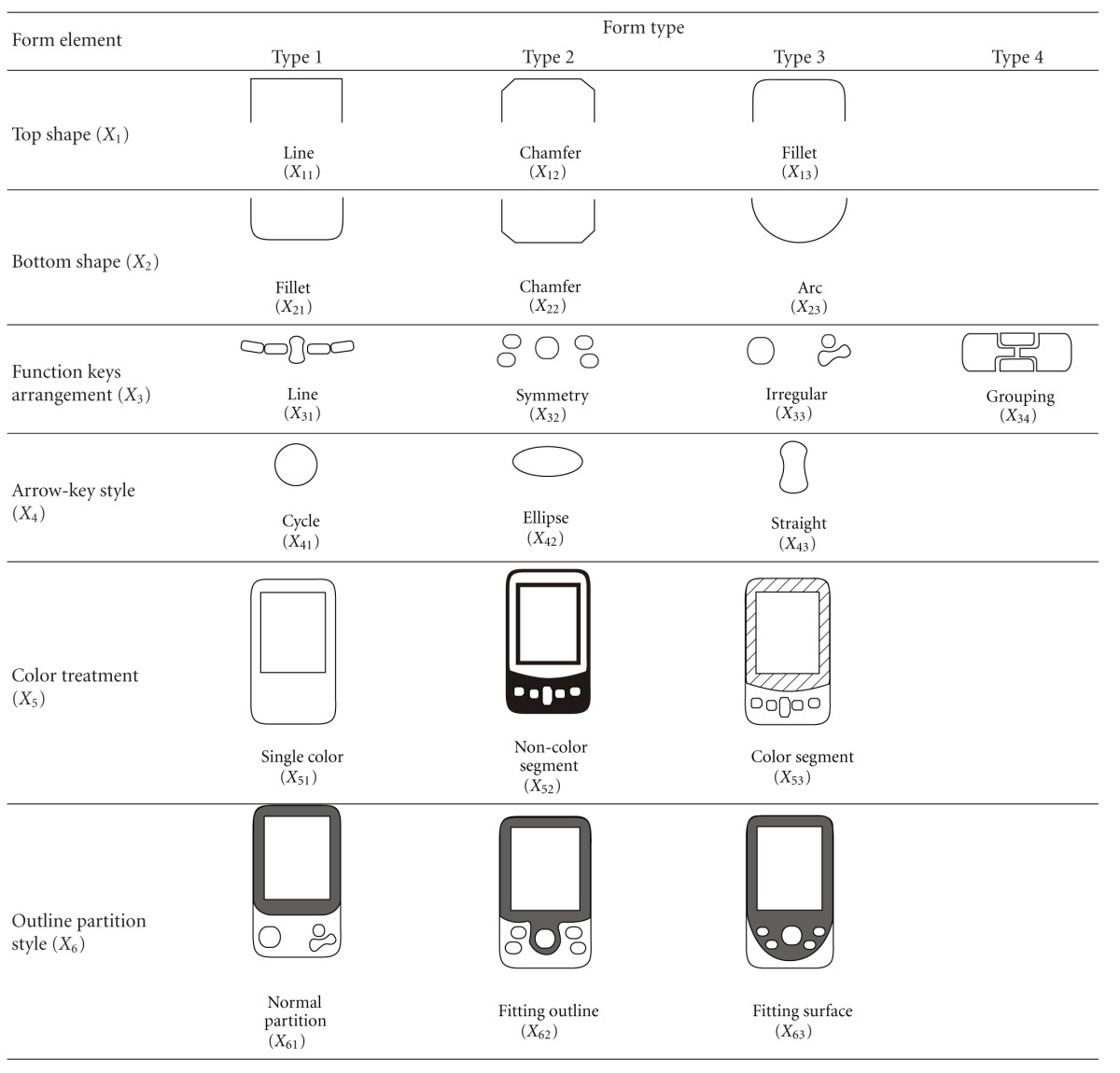

**Table 2:** Product image assessments of 30 PDA samples.

PDA no.	X_1_	X_2_	X_3_	X_4_	X_5_	X_6_	S-C value
1	3	1	1	3	1	1	1.67
2	3	1	1	3	1	1	2.33
3	2	2	3	3	3	2	3.33
4	1	2	2	3	1	1	3.67
5	3	3	1	2	1	1	1.67
6	2	2	1	1	1	1	8.33
7	3	3	1	3	1	3	2.33
8	3	3	2	2	1	1	2.33
9	3	3	2	2	2	1	6.33
10	3	1	3	1	2	1	3.33
11	3	3	2	2	2	1	4.67
12	1	3	1	1	1	1	1.67
13	3	3	1	1	1	1	3.33
14	2	1	1	2	3	2	2.33
15	3	1	2	1	3	3	2.33
16	3	1	3	1	3	3	4.67
17	2	3	2	1	2	1	7.33
18	1	3	2	2	2	2	8.33
19	3	3	1	2	1	1	4.67
20	3	2	4	3	1	1	1.67
21	3	1	1	2	1	1	5.67
22	2	3	1	1	1	1	1.67
23	2	2	2	1	2	1	1.33
24	3	3	4	2	3	1	4.67
25*	3	1	2	2	3	2	5.33
26*	2	2	1	2	1	1	2.33
27*	3	3	1	1	1	1	4.33
28*	3	2	2	1	1	1	5.67
29*	2	1	1	1	1	1	2.33
30*	3	3	1	3	2	2	4.33

*Mean that the 6 PDA samples are the test set for quantitative analysis models.

**Table 3:** The result of QTTI analysis.

Form element		Form type		Category grade (form type grade)		Partial correlation coefficient
				Complex	Simple	
		*X*_11_	Line		1.00	
*X*_1_	Top shape	*X*_12_	Chamfer		0.54	0.26
		*X*_13_	Fillet	−0.42		

		*X*_21_	Fillet	−0.11		
*X*_2_	Bottom shape	*X*_22_	Chamfer		0.61	0.14
		*X*_23_	Arc	−0.19		

		*X*_31_	Line		0.01	
*X*_3_	Function-keys arrangement	*X*_32_	Symmetry	−0.37		0.16
		*X*_33_	Irregular		0.65	
		*X*_34_	Grouping		0.48	

		*X*_41_	Cycle	−0.42		
*X*_4_	Arrow-key style	*X*_42_	Ellipse		1.28	0.42
		*X*_43_	Straight	−1.29		

		*X*_51_	Single color	−0.21		
*X*_5_	Color treatment	*X*_52_	Noncolor segment		1.35	0.37
		*X*_53_	Color segment	−1.06		

		*X*_61_	Normal partition	−0.20		
*X*_6_	Outline partition style	*X*_62_	Fitting outline	−0.13		0.23
		*X*_63_	Fitting surface		1.32	

Constant = 3.74, *R* = 0.55, *R* ^2^ = 0.31.

**Table 4:** Neurons of two NN models.

	Input layer: 6 neurons, including six form elements of PDAs
NN-FE model	Hidden layer: 4 neurons, (6 + 1)/2 = 3.5 *≒* 4
	Output layer: 1 neuron for the S-C image value

	Input layer: 19 neurons, including 19 types of six form elements
NN-FT model	Hidden layer: 10 neurons, (19 + 1)/2 = 10
	Output layer: 1 neuron for the S-C image value

**Table 5:** Predicted image values and RMSE of four models for the test.

PDA no.	25	26	27	28	29	30	RMSE
Subject Assessment	5.33	2.33	4.33	5.67	2.33	4.33	
QTTI	2.93	5.77	2.31	2.73	3.35	3.07	0.2343
GP	2.85	1.54	0.34	0.05	1.16	2.43	0.3143
NN-FE	5.08	6.28	3.57	4.18	5.32	8.22	0.2663
NN-FT	2.50	8.27	3.44	6.04	3.97	2.69	0.2875

**Table 6:** Neurons, learning rate, and momentum of NN models.

	Input neuron	Hidden neuron	Output neuron	Learning rate	Momentum	Note
NN-FE-P	6	4	1	0.2	0.5	According to our previous study
NN-FE-S	6	4	1	0.9	0.6	Research issue is very simple
NN-FE-C	6	4	1	0.1	0.1	Research issue is more complicated
NN-FE-N	6	4	1	0.05	0.5	Research issue is complex and very noisy
NN-FT-P	19	10	1	0.2	0.5	According to our previous study
NN-FT-S	19	10	1	0.9	0.6	Research issue is very simple
NN-FT-C	19	10	1	0.1	0.1	Research issue is more complicated
NN-FT-N	19	10	1	0.05	0.5	Research issue is complex and very noisy

**Table 7:** Predicted image values and RMSE of NN models for the test set.

PDA no.	25	26	27	28	29	30	RMSE	
NN-FE-P	5.08	6.28	3.57	4.18	5.32	8.22	0.2663	0.3033
NN-FE-S	4.43	5.04	3.53	5.20	9.23	8.76	0.3565	
NN-FE-C	1.45	2.95	3.71	9.03	9.38	6.65	0.3701	
NN-FE-N	4.63	6.04	3.26	3.67	5.25	5.41	0.2203	

NN-FT-P	2.50	8.27	3.44	6.04	3.97	2.69	0.2875	0.3168
NN-FT-S	1.58	8.27	3.41	1.46	3.38	5.07	0.3405	
NN-FT-C	2.22	8.19	3.42	7.03	3.72	3.46	0.2865	
NN-FT-N	1.49	8.39	3.40	2.08	3.83	1.66	0.3526	

**Table 8:** The design support information for product form elements of PDAs.

Form element		With “Simple” image		With “Complex” image	
*X*_1_	Top shape	*X*_11_	Line	*X*_13_	Fillet
		*X*_12_	Chamfer		

*X*_2_	Bottom shape	*X*_22_	Chamfer	*X*_23_	Arc
				*X*_21_	Fillet

*X*_3_	Function-keys arrangement	*X*_33_	Irregular	*X*_32_	Symmetry
		*X*_34_	Grouping		
		*X*_31_	Line		

*X*_4_	Arrow-key style	*X*_42_	Ellipse	*X*_43_	Straight
				*X*_41_	Cycle

*X*_5_	Color treatment	*X*_52_	Noncolor segment	*X*_53_	Color segment
				*X*_51_	Single color

*X*_6_	Outline partition style	*X*_63_	Fitting surface	*X*_61_	Normal partition
				*X*_62_	Fitting outline
